# Severe influenza A viral pneumonia in a hemodialysis patient: successful treatment with steroid pulse therapy

**DOI:** 10.1007/s13730-024-00951-6

**Published:** 2024-12-11

**Authors:** Hiroki Ito, Sadatoshi Ito, Takuo Hirose, Tomoyoshi Kimura, Takefumi Mori, Sadayoshi Ito

**Affiliations:** 1 Division of Nephrology and Endocrinology, Faculty of Medicine, Tohoku Medical and Pharmaceutical University, 1-15-1 Fukumuro, Miyagino-Ku, Sendai, Miyagi 983-8536 Japan; 2 Katta General Hospital, Shiroishi, Japan; 3 Division of Integrative Renal Replacement Therapy, Faculty of Medicine, Tohoku Medical and Pharmaceutical University, Sendai, Japan; 4 Division of Nephrology, Endocrinology and Vascular Medicine, Graduate School of Medicine, Tohoku University School of Medicine, Sendai, Japan

**Keywords:** Seasonal influenza, Hemodialysis, Steroid pulse therapy

## Abstract

Seasonal influenza is prevalent globally, particularly during winter months. It is well documented that this disease causes severe, often fatal complications in hemodialysis patients. While numerous reports have focused on novel influenza viruses, there is a paucity of case reports detailing seasonal influenza viral infections in this patient population. This case presents a 71-year-old male undergoing hemodialysis who developed severe seasonal influenza A pneumonia despite receiving the influenza vaccine and early antiviral treatment. Initially presenting with fever, cough, and myalgia, the patient was diagnosed with influenza A virus infection and hospitalized due to heightened risk associated with dialysis and an elevated inflammatory response. Despite treatment with two different antiviral medications, his condition deteriorated, leading to ARDS (acute respiratory distress syndrome). The administration of steroid pulse therapy resulted in significant clinical improvement. This case underscores the severe nature of influenza virus-related illnesses in dialysis patients, even with vaccination and early antiviral intervention. It also suggests the potential benefit of early steroid pulse therapy in managing severe influenza pneumonia in high-risk individuals.

## Introduction

Seasonal influenza virus infection poses a significant health risk to the general population, with particular concern for high-risk groups such as hemodialysis patients [1]. These individuals are more susceptible to severe complications due to impaired innate and adaptive immune systems, including defects in complement activation, B- and T-cell function, and underlying comorbidities [1–5]. Despite standard preventive measures like vaccination and antiviral therapies, their efficacy can be limited in this population [6–10]. Hemodialysis patients often present with atypical influenza symptoms, leading to delayed diagnosis and treatment [11]. Current guidelines primarily focus on vaccination and early antiviral treatment for influenza management in hemodialysis patients [12, 13]. However, there is a paucity of case reports detailing the management of severe cases despite these interventions.

This case report presents a hemodialysis patient who developed severe influenza A pneumonia despite vaccination and prompt antiviral therapy, but responded positively to steroid pulse therapy. We aim to contribute to the understanding of the clinical course and management of severe influenza infections in hemodialysis patients. We hope this report offers valuable insights for clinicians managing similar challenging cases in this high-risk population.

## Case report

A 71-year-old male patient with kidney failure undergoing hemodialysis was admitted to our hospital with a 2-day history of fever, cough, and arthralgia. The patient initiated hemodialysis at age 61 due to diabetic nephropathy. His medical history included lumbar disc herniation (age 20), hypertension, type 2 diabetes mellitus (age 56), cerebral infarction (age 60), secondary hyperparathyroidism (treated pharmacologically since dialysis initiation), and peripheral arterial disease with left femoral-popliteal artery bypass surgery (age 61). At age 66, he experienced hospitalization for bacterial pneumonia and lower-limb cellulitis. The patient received vonoprazan (10 mg/day), nifedipine (40 mg/day), linagliptin (5 mg/day), clopidogrel (75 mg/day), carvedilol (2.5 mg/day), and calcium carbonate (2000 mg/day). He had received a seasonal influenza vaccine approximately 3 months prior. Notably, his sister, who visited 2 days before symptom onset, was diagnosed with influenza A infection. The patient was subsequently diagnosed with influenza A infection via immunochromatography after exposure to his sister. A single inhalation dose of 40 mg laninamivir octanoate hydrate was prescribed. However, the following day, the patient developed a fever of 39.0 °C, severe malaise, and difficulty moving, necessitating urgent transport to the emergency department.

**Fig. 1:**
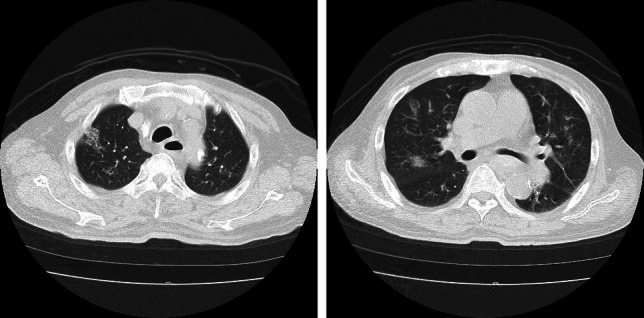
A computed tomography scan on the first day of hospitalization shows scattered ground-glass opacities in both lungs without pleural effusion

On admission, the patient’s vital signs were as follows: temperature 39.0 °C, blood pressure 112/60 mmHg, heart rate 108 bpm, and oxygen saturation 96% on room air. Initial blood and biochemical tests, limited to emergency department capabilities at night, revealed elevated inflammatory markers (white blood cell count 12.7 × 10^3^ /μL, C-reactive protein 23.0 mg/dL), and anemia (red blood cell count 12.7 × 10^6^ /μL, hemoglobin 10.3 g/dL, platelet count 16.1 × 10^3^ /μL). Electrolyte levels were within normal limits except for hyponatremia (sodium 134 mEq/L). Liver function tests, renal function tests, and other biochemical parameters were unremarkable, with the exception of elevated creatinine kinase (374 IU/L, albumin 3.2 g/dL, total protein 5.7 g/dL, γGTP 22 IU/L, aspartate aminotransferase 20 IU/L, alanine transaminase 11 IU/L, glucose 179 mg/dL, total bilirubin 1.4 mg/dL, blood urea nitrogen 36 mg/dL, creatinine 6.7 mg/dL, amylase 51 IU/L). Chest computed tomography (CT) demonstrated subtle interstitial lung changes bilaterally, without pleural effusion (Fig. 1). Echocardiography showed an ejection fraction of approximately 60% with inferior vena cava collapse, indicating no fluid overload. Electrocardiography revealed sinus tachycardia without significant changes from previous recordings. Rapid diagnostic tests for COVID-19, *Streptococcus pneumoniae*, and *Legionella* were negative.

**Fig. 2:**
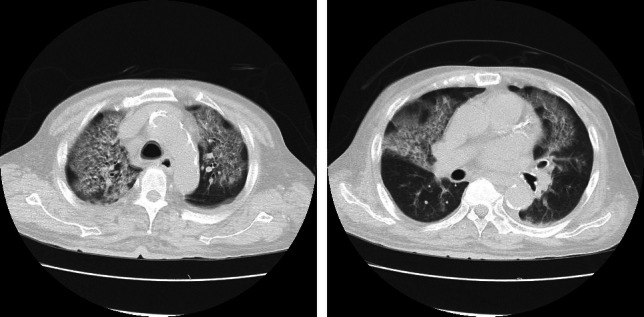
A computed tomography scan on day 3 of hospitalization shows extensive ground-glass opacities in all lung lobes

CT findings suggested the possibility of atypical pneumonia in addition to influenza A infection. Consequently, sputum and blood cultures were obtained, and treatment with peramivir (100 mg) and moxifloxacin (400 mg/day) was initiated. The patient’s respiratory condition deteriorated 2 days post-admission, with oxygen saturation dropping to 90% (PaO_2_ 62 Torr) despite high-flow oxygen therapy with a reservoir mask delivering 10 L/min. A repeat CT scan revealed extensive bilateral ground-glass opacities, consistent with acute respiratory distress syndrome (ARDS) secondary to severe influenza viral pneumonia (Fig. 2).

**Fig. 3:**
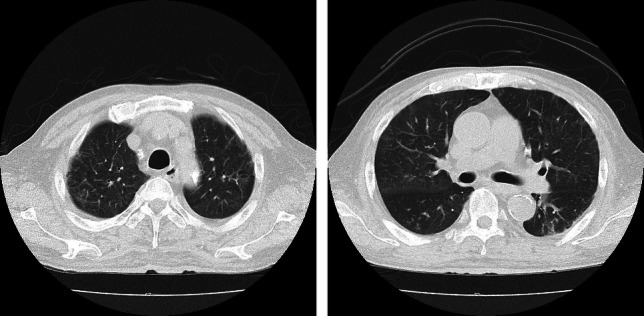
A computed tomography scan on day 10 of hospitalization shows resolution of ground-glass opacities

Steroid pulse therapy with methylprednisolone (500 mg/day) for 3 days, followed by oral prednisolone (40 mg/day), resulted in improved respiratory status, resolution of lung opacities (Fig. 3), and decreased inflammation. Steroid dosage was gradually reduced over 10 days. Throughout the course of the disease, dry weight could not be reduced and thus remained unchanged due to severe hypotension during dialysis, a direct consequence of the patient’s critical condition. Cultures remained negative for bacterial pathogens, supporting the diagnosis of severe influenza A pneumonia without superinfection. The patient had a history of cerebral infarction at age 60, but had no residual deficits and was fully independent in his activities of daily living. He had never experienced aspiration pneumonia since the stroke. In this case, while we considered the possibility of aspiration pneumonia given his history, the rapid onset of symptoms, the confirmed influenza A infection, and the characteristic radiographic findings were more consistent with viral pneumonia. Additionally, the patient had no recent history of dysphagia or aspiration events. Based on these factors, we concluded that viral pneumonia was more likely in this case. Despite the absence of polymerase chain reaction testing for the influenza virus, contemporary surveillance data confirmed the circulation of A/H1N1pdm and H3N2 influenza A subtypes without novel strains. The patient was discharged on the 16th hospital day.

## Discussion

This case report presents a hemodialysis patient who developed severe influenza A pneumonia despite receiving the seasonal influenza vaccine. Individuals with kidney failure are at an elevated risk for influenza complications due to immune dysfunction, advanced age, and comorbidities such as diabetes mellitus, malnutrition, invasive dialysis procedures, disruption of skin and mucosa barriers, and nosocomial infections[1, 14]. The underlying immunodeficiency in kidney failure patients involves impaired complement activation and deficiencies in neutrophil, monocyte/macrophage, and T-cell and B-cell function [5]. While vaccination is crucial for hemodialysis patients, its efficacy may be compromised by uremia-induced immune dysfunction [15, 16]. Nevertheless, vaccination remains strongly recommended due to the elevated influenza risk in this population [17].

Initial treatment with laninamivir was unsuccessful, necessitating the addition of peramivir, emphasizing the challenges in managing this population. Given the patient’s high-risk status as a hemodialysis recipient, the rapid deterioration of his condition, and the potential for atypical pneumonia, we initiated a combination therapy. Peramivir (100 mg) was chosen for its potent antiviral activity against influenza, while moxifloxacin (400 mg/day) was added to cover potential bacterial co-infection, including atypical pathogens, which is common in severe influenza cases, especially in immunocompromised patients.

The use of corticosteroids in viral pneumonia, particularly in influenza cases, remains a subject of ongoing debate in the medical community. Several studies have produced conflicting results regarding the effectiveness of steroid pulse therapy in treating viral infections or ARDS [18–20]. Some research suggests that corticosteroid use may be associated with prolonged viral shedding and an increased risk of secondary infections, potentially worsening outcomes [21]. However, recent meta-analyses have shown potential benefits in severe cases, particularly when administered early in the course of ARDS [18]. In our case, the decision to use steroid pulse therapy was based on the patient’s rapid deterioration despite antiviral treatment and the development of ARDS. We carefully weighed the potential risks and benefits, considering the patient’s individual clinical context [22].

Our patient’s initial presentation without typical upper respiratory symptoms highlights the potential for atypical manifestations of influenza in hemodialysis patients. This can lead to diagnostic delays and emphasizes the necessity of maintaining a high level of suspicion for influenza amongst this population, particularly during peak seasons [11]. Comprehensive care, including management of comorbidities, is essential for hemodialysis patients [23]. A multidisciplinary approach is crucial for optimal care of these complex patients. Additionally, strict infection control measures are necessary to prevent nosocomial transmission within dialysis units [24].

This case highlights the potential for severe complications from seasonal influenza in hemodialysis patients, despite vaccination and prompt antiviral treatment. Our findings suggest that these patients may have a suboptimal response to initial antiviral therapy, emphasizing the need for close monitoring and potential treatment escalation. In this case, the patient’s condition deteriorated rapidly within 48 h of initiating antiviral therapy, which is generally considered an adequate time frame to observe initial treatment response in influenza cases [25, 26]. This rapid progression, despite early intervention, highlights the potential for severe complications in hemodialysis patients.

## Limitaitons

This case report has two main limitations. First, while we attribute the patient’s improvement to steroid pulse therapy, we acknowledge the importance of considering alternative explanations for the observed recovery. We carefully evaluated the possibility of adrenal insufficiency, secondary bacterial infection, negative pressure pulmonary edema, and afterload mismatch. Adrenal insufficiency was unlikely given the patient’s clinical presentation and rapid response to standard steroid dosing. The patient was not taking any medications known to induce adrenal insufficiency, had no hypotension, and had not experienced fatigue, weakness, or gastrointestinal symptoms typical of adrenal insufficiency. While electrolyte abnormalities might have been corrected by dialysis, there were no significant electrolyte imbalances noted, and no skin hyperpigmentation was observed [27]. Sputum and blood cultures remained negative, ruling out secondary bacterial infection. Negative pressure pulmonary edema was deemed improbable as the patient remained fully conscious throughout the course of illness, never complained of choking sensations, and had no episodes suggestive of upper airway obstruction[28]. The patient’s hemodynamic stability did not support significant afterload mismatch. Throughout the treatment, the patient’s blood pressure ranged from 106 to 164 mmHg systolic and 54 to 82 mmHg diastolic, which is not indicative of hemodynamics typically associated with afterload mismatch [29]. The second limitation is that this report describes a single case, which limits the generalizability of our findings. While the temporal relationship between steroid administration and clinical improvement strengthens our hypothesis regarding the efficacy of steroid pulse therapy in this case, further research involving a larger number of patients is necessary to establish the broader applicability of this treatment approach in hemodialysis patients with severe influenza.
